# A Workshop on Cognitive Aging and Impairment in the 9/11-Exposed Population

**DOI:** 10.3390/ijerph18020681

**Published:** 2021-01-14

**Authors:** Robert D. Daniels, Sean A. P. Clouston, Charles B. Hall, Kristi R. Anderson, David A. Bennett, Evelyn J. Bromet, Geoffrey M. Calvert, Tania Carreón, Steven T. DeKosky, Erica D. Diminich, Caleb E. Finch, Sam Gandy, William C. Kreisl, Minos Kritikos, Travis L. Kubale, Michelle M. Mielke, Elaine R. Peskind, Murray A. Raskind, Marcus Richards, Mary Sano, Albeliz Santiago-Colón, Richard P. Sloan, Avron Spiro, Neil Vasdev, Benjamin J. Luft, Dori B. Reissman

**Affiliations:** 1 World Trade Center Health Program, Centers for Disease Control and Prevention, National Institute for Occupational Safety and Health, Washington, DC 20201, USA; kga7@cdc.gov (K.R.A.); jac6@cdc.gov (G.M.C.); tjc5@cdc.gov (T.C.); tek2@cdc.gov (T.L.K.); ASantiagoColon@cdc.gov (A.S.-C.); dvs7@cdc.gov (D.B.R.); 2 Renaissance School of Medicine, Stony Brook University, Stony Brook, NY 11794, USA; Sean.Clouston@stonybrookmedicine.edu (S.A.P.C.); Evelyn.Bromet@stonybrookmedicine.edu (E.J.B.); Erica.Diminich@stonybrookmedicine.edu (E.D.D.); minos.kritikos@stonybrookmedicine.edu (M.K.); Benjamin.Luft@stonybrookmedicine.edu (B.J.L.); 3 Department of Epidemiology & Population Health (Biostatistics), Albert Einstein College of Medicine, Bronx, NY 10461, USA; charles.hall@einsteinmed.org; 4 Department of Neurological Sciences, Rush Medical College, Rush University, Chicago, IL 60612, USA; David_A_Bennett@rush.edu; 5 McKnight Brain Institute, University of Florida, Gainesville, FL 32611, USA; Steven.DeKosky@neurology.ufl.edu; 6 USC Leonard Davis School of Gerontology, Los Angeles, CA 90089, USA; cefinch@usc.edu; 7 Icahn School of Medicine at Mount Sinai, New York, NY 10029, USA; samuel.gandy@mssm.edu (S.G.); mary.sano@mssm.edu (M.S.); 8 Taub Institute for Research on Alzheimer’s Disease and the Aging Brain, New York, NY 10032, USA; wck2107@cumc.columbia.edu; 9 Division of Epidemiology and Department of Neurology, Department of Health Sciences Research, Mayo Clinic, Rochester, MN 55905, USA; Mielke.Michelle@mayo.edu; 10 Department of Psychiatry and Behavioral Sciences, University of Washington School of Medicine, Seattle, WA 98195, USA; peskind@uw.edu; 11 Northwest Mental Illness Research, Education and Clinical Center (MIRECC), VA Puget Sound Health Care System, Seattle, WA 98108, USA; Murray.Raskind@va.gov; 12 Faculty of Population Health Sciences, University College London, London WC1E 6BT, UK; m.richards@ucl.ac.uk; 13 Division of Behavioral Medicine, Columbia University, New York, NY 10027, USA; rps7@cumc.columbia.edu; 14 Boston University Schools of Public Health and Medicine and Veterans Affairs Boston Healthcare System, Boston, MA 02130, USA; aspiro3@bu.edu; 15 Azrieli Centre for Neuro-Radiochemistry, Brain Health Imaging Centre, Centre for Addiction and Mental Health (CAMH) & Department of Psychiatry, University of Toronto, Toronto, ON M5S, Canada; neil.vasdev@utoronto.ca

**Keywords:** World Trade Center Health Program, 9/11, mild cognitive impairment, emerging medical conditions, disaster epidemiology, review

## Abstract

The terrorist attacks on 11 September 2001 potentially exposed more than 400,000 responders, workers, and residents to psychological and physical stressors, and numerous hazardous pollutants. In 2011, the World Trade Center Health Program (WTCHP) was mandated to monitor and treat persons with 9/11-related adverse health conditions and conduct research on physical and mental health conditions related to the attacks. Emerging evidence suggests that persons exposed to 9/11 may be at increased risk of developing mild cognitive impairment. To investigate further, the WTCHP convened a scientific workshop that examined the natural history of cognitive aging and impairment, biomarkers in the pathway of neurodegenerative diseases, the neuropathological changes associated with hazardous exposures, and the evidence of cognitive decline and impairment in the 9/11-exposed population. Invited participants included scientists actively involved in health-effects research of 9/11-exposed persons and other at-risk populations. Attendees shared relevant research results from their respective programs and discussed several options for enhancements to research and surveillance activities, including the development of a multi-institutional collaborative research network. The goal of this report is to outline the meeting’s agenda and provide an overview of the presentation materials and group discussion.

## 1. Introduction

The 11 September 2001 terrorist attacks on the World Trade Center (WTC) potentially exposed over 400,000 responders, workers, and residents to an array of psychological and physical stressors, as well as numerous potentially hazardous pollutants. Nearly two decades later, there is emerging evidence suggesting that 9/11 exposures may increase the risk of experiencing mild cognitive impairment (MCI) [1,2,3,4,5]. Operational definitions of MCI differ; however, it is generally described as an intermediate clinical state between normal cognitive function and dementia [6]. Patients with MCI are at increased risk of Alzheimer’s disease (AD) and other dementias. Causal mechanisms for the association of 9/11 exposure with MCI are unclear; however, two potential pathways have been suggested. First, unremitting and chronic symptoms of WTC-related post-traumatic stress disorder (PTSD) among individuals who experienced the attacks, or participated in the subsequent rescue, recovery and rehabilitation efforts may by causally associated with the development of MCI. This pathway is supported by previous studies of combat veterans that found associations between PTSD and cognitive impairment [7,8]. Similar results have been reported in recent studies of 9/11 first responders [1,3,5] and there is a relatively large and persistent exposure-dependent burden of PTSD in the 9/11-exposed population [9]. Second, environmental exposures to airborne neurotoxins and fine particulates that were generated by the collapsing towers and subsequent fires may be associated with MCI. This cause is supported by evidence suggesting that inhalation of air pollutants can increase the risk of neurodegenerative diseases [10,11,12,13,14,15,16,17]. Studies have found exposure–response associations between exposure to dust and debris from the collapsing towers and measures of cognitive functioning [2,3].

The federally-mandated World Trade Center Health Program (WTCHP) is a longstanding health surveillance, treatment and research program aimed to improve the care and well-being of the affected population [18]. The WTCHP is seeking improvements to MCI surveillance and research to characterize its potential health burden, elucidate causal pathways, mitigate poor outcomes in those initially unaffected, and subsequently improve the quality of life and treatment of persons adversely affected by the terror attacks. To gather information, a scientific workshop was convened to examine the natural history of cognitive aging and impairment, biomarkers in the pathway of neurodegenerative diseases, the neuropathological changes associated with hazardous exposures, and current evidence of cognitive decline and impairment in the 9/11-exposed population. This report describes the information and individual perspectives presented in each workshop listening session. The exchange of this information is intended to enhance efforts addressing cognitive decline in WTCHP members and other at-risk populations.

## 2. Materials and Methods

The workshop was held in Alexandria, Virginia from 29 to 30 October 2019. Invited participants included clinicians, epidemiologists, WTCHP researchers, and other experts actively involved in cognitive impairment studies. The aims of the workshop were to review the science on MCI, weigh the evidence of increased risk in the 9/11-exposed population, identify research gaps, and discuss potential risk management strategies, including surveillance and treatment options. The focus was to glean perspectives from individual workshop participants; therefore, there was no charge or encouragement to reach consensus in recommendations. The workshop centered on answering four key program questions:

1. What is the evidence of causal associations between 9/11 exposure, PTSD, and the risk of adverse cognitive function?
2. What is the potential health burden from MCI?
3. What are the research gaps?
4. What steps are needed to manage MCI risks and improve care?

Planning was accomplished jointly by researchers who were actively involved in studies of cognition in the 9/11-exposed population and WTCHP staff. The list of invited attendees and workshop agenda were structured to ensure available expertise to lead discussions in the six listening sessions described in Table 1. To stimulate discussion, participants were provided a list of questions beforehand that were intended to be addressed in each session (Table 1). All sessions were recorded and transcribed for the record. The slides and transcripts were used to develop this report, which presents an overview of workshop, the exchange of facts and information on cognitive decline and other issues of aging, as well as individual perspectives on future research. The report also discusses ongoing WTCHP initiatives. The research in this area is evolving; therefore, session discussions were augmented by relevant studies published since the workshop convened, based on input from workshop participants made during manuscript development.

## 3. Results

### 3.1. Session I: The Natural History of Cognitive Aging and Impairment

Session presenters: Dr. Richards, Dr. Sano, and Dr. Bennett. Moderators: Dr. Clouston and Dr. Hall.

Key Points:

- Both dementia and MCI represent clinically observed significant declines in previously addressed cognitive function; the primary difference being impairment severity.
- The prevalence of MCI is significant, ranging from 4% to 19% of persons aged 65 or older.
- Approximately 40% of dementia is believed attributable to twelve modifiable risk factors: lower educational attainment, hypertension, obesity, hearing loss, depression, diabetes, physical inactivity, smoking, social isolation, traumatic brain injury, excess alcohol consumption, and air pollution.
- Causes of MCI are varied and have different impacts (e.g., clinical profile, trajectory of decline, interaction with neurodegenerative disease, and success of preventions and interventions).
- The effects of MCI and/or dementia on cognitive domains overlap, making it difficult to robustly define patterns of cognition.

Cognitive decline is a common characteristic of aging that is likely caused by complex cascades of events occurring over time. The rate and magnitude of the decline are highly variable among individuals, with some showing cognitive stability throughout life, others exhibiting mild impairment (e.g., MCI), still others rapidly progressing to dementia. The three most common causes of dementia in old age are AD, cerebrovascular disease, and Lewy body disease (LBD). Yet these pathologies only explain approximately 41% of the variation in cognitive decline [19].

Given similarities, most of the literature describes the life course of MCI as a component of the natural history of dementia. Both dementia and MCI are characterized by a decline in a previously attained cognitive level. The primary difference between the two disorders is severity of impairment in activities of daily living, whereby those with MCI continue to be high functioning while those with dementia are debilitated. MCI usually precedes dementia; however, not all MCI patients will progress to dementia and not all dementia patients have a previous diagnosis of MCI. Some individuals diagnosed with MCI at baseline revert to normal cognition, with reported revision rates ranging from 4% to 59% [20]; however, these individuals are at increased risk for transitioning back to MCI or progression to dementia [21,22]. Multiple studies suggest that the rate of conversion to AD appears highest in amnestic MCI (aMCI), ranging between 10 and 15% per year [23]. Worldwide, nearly 50 million people are living with dementia. As the world population ages, this number is expected to increase [24].

MCI is a condition describing a transitional area of the continuum between normal cognitive function and dementia. MCI is much more common than dementia, with prevalence in persons aged 65 or older estimated to range from 4% to 19% [24]. MCI definitions are evolving, and diagnostic criteria have varied; however, they generally require some measurable cognitive deficit that allows for a clinical classification (a clinician’s judgement) of impairment at a level below the diagnostic criteria for dementia. Measuring cognitive decline is difficult given that the best evidence is longitudinal, by definition. Clinical presentation of MCI is heterogeneous, whereby cognitive impairment may be amnestic, or can involve a single non-memory domain (e.g., language, executive function, visuospatial skills), or multiple cognitive domains. Etiology is also varied, with links to neurodegenerative, traumatic, psychiatric (e.g., depression), metabolic, vascular, genetic, and environmental causes, among others. There is less literature describing modifiable risk factors for MCI compared to dementia; however, there is a general consistency in factors between the two outcomes. The most recent model by Livingston et al. (2020) predicts that approximately 40% of dementia is attributable to a combination of twelve modifiable risk factors that differ by life course: less education in earlier life (age <18 years); hearing loss, hypertension, traumatic brain injury, excessive alcohol consumption, and obesity in midlife (age 45–65 years); and smoking, depression, physical inactivity, social isolation, diabetes, and air pollution in later life (age >65 years). The remaining 60% of risk is attributable to a combination of other potential modifiable factors (e.g., sleep and hypercholesterolemia) and non-modifiable factors, such as a genetic predisposition (e.g., the Apolipoprotein E ε4 genotype (APOE4) is estimated to account for 7% of the attributable risk). These factors may not act independently or directly, thus adding to the complexity of causal pathways. Many factors are largely influenced by societal and economic stressors; therefore, effective prevention likely requires both health promotion and societal action.

### 3.2. Session II: Novel Identifiable Markers in the Pathway of Neurodegenerative Disease

Session presenters: Dr. Kreisl, Dr. Vasdev, Dr. Mielke, and Dr. Gandy. Moderator: Dr. Luft.

Key Points:

- A framework comprising imaging and cerebrospinal fluid (CSF) biomarkers that are grouped by three pathologic processes of amyloid, tauopathy, and neurodegeneration/injury is recommended for brain aging research.
- Positron emission tomography (PET) has been used to measure neuroinflammation, a proposed pathogenic contributor to Alzheimer’s disease and other cognitive disorders, but only one reliable biomarker has been identified thus far.
- Emerging plasma or serum-based biomarkers offer a lower-cost, less-invasive alternative to imaging and CSF. These peripheral markers provide a measure of severity but do not indicate the location of the effect.

#### 3.2.1. Neuroimaging

Neuroimaging is an essential part of evaluating neurodegeneration in MCI and dementia patients. Brain imaging can identify specific causes of decline; thus, it can have a role in differential diagnosis. Neuroimaging also enables evaluating disease progression and can elucidate neuropathology. Structural neuroimaging with magnetic resonance imaging (MRI) has been used to examine markers of neurodegeneration that are characteristic of progression from normal aging to MCI and from MCI to clinical AD dementia [25]. These evaluations have focused on markers of regional brain volume loss, such as atrophy of the hippocampus or entorhinal cortex. There is also evidence that deficits in cerebral blood flow, as measured by single-photon emission computed tomography (SPECT), and cerebral glucose metabolism, as measured by fluorodeoxyglucose (FDG)-positron emission tomography (PET), are predictive of future development of AD in individuals with MCI [25].

#### 3.2.2. Biomarker Research Framework

There is a set of core imaging and cerebrospinal fluid (CSF) biomarkers that have been clinically validated and are predominantly used in brain aging research. To date, biomarker development has centered on neuropathologic changes considered important to AD, such as β-amyloid deposition, tau hyperphosphorylation, and neurodegeneration. These biomarkers may also be useful in understanding MCI, given its position on the continuum between normal cognition and dementia. In 2018, the National Institute on Aging (NIA) and the Alzheimer’s Association (AA) adopted a research framework that focuses on the diagnosis of AD as a biological construct using in vivo biomarkers [26]. Biomarkers were grouped into those for β amyloid deposition, aggregated tau, and neurodegeneration or neuronal injury (i.e., the NIA-AA AT(N) biomarker model, Table 2). The diagnostic criteria established by the NIA-AA AT(N) framework is an important departure from subjective and objective cognitive assessments. As such, its widespread use as sole diagnostic criteria for AD is controversial [27].

This AT(N) classification scheme recognizes three general groups of biomarkers based on the nature of the pathologic process measured by each of them [26]. There are CSF and imaging biomarkers in each group; therefore, AT(N) biomarker characterization can be completed by either or both marker types. For example, the amyloid biomarkers involving Aβ-binding positron emission tomography (PET) ligands and Aβ42 in CSF are considered interchangeable [28]. In general, there is sufficient evidence that the presence of amyloid and tau, measured either by PET or in CSF, indicate increased risk of cognitive impairment in older adults. There are also longitudinal data indicating that CSF concentrations of total tau, phosphorylated tau181 (P-tau181), and Aβ42 are associated with future development of AD dementia in patients with MCI [29,30]. However, these findings are tempered by the fact that many cognitively normal people with these markers do not develop dementia within a clinically relevant timeframe [31]. Existing biomarkers would benefit from efforts to standardize methodologies and validate biomarkers in larger and more heterogeneous populations. Longitudinal changes in biomarkers over the disease course also need to be evaluated. More research is needed to elucidate conditions and combinations of biomarkers that maximize sensitivity and specificity.

#### 3.2.3. Other Potential Biomarkers

The AT(N) classification system allows for adding new biomarkers within existing AT(N) groups. It is also possible that new groups may be added to the framework in the future. For example, newly developed biomarkers for cerebrovascular disease may comprise a new group (V) supporting a more rigorous ATV(N) classification system [26]. Other promising biomarkers include blood-based markers of amyloid, tau, and neurodegeneration and markers of neuroinflammation, both of which are described in Section 3.2.3.

Recent advances in ultrasensitive measurement technologies (e.g., immunomagnetic reduction, single-molecule array, and immunoprecipitation mass spectrometry) have rapidly advanced the use of blood-based biomarkers for neurodegenerative diseases. Compared to core AT(N) biomarkers, plasma- and serum-based biomarkers have the general advantage of lower costs, less invasiveness, and wider availability in many settings [31]. These peripheral markers may serve as screening tools in primary care and may provide efficient monitoring of disease progression. Blood-based biomarkers plausibly fitting under the AT(N) research framework include amyloid, Ptau 181, total tau, and neurofilament light chain (NfL) [32,33,34,35,36,37,38]. For example, a recent study using immunomagnetic reduction to evaluate levels of plasma biomarkers found that higher levels of Aβ1-42 were correlated with cognitive deterioration in aMCI patients [35]. A similar study found that higher levels of Aβ42 and total tau in aMCI patients were associated with lower episodic verbal memory performance at baseline and cognitive decline over the course of follow up (average follow-up period of 1.5 years) [37]. The product of Aβ42 and total tau offered a greater differential value. Plasma measures of P-tau181 are also now available and have been shown to differ by clinical status, correlate with both amyloid and tau PET findings, and predict rate of cognitive decline [39,40,41]. Other studies have found that plasma NfL level, a general marker of neurodegeneration, was associated with AD and MCI, and correlated with future progression of cognitive decline, brain atrophy, and brain hypometabolism [33,38,42]. Overall, there is growing evidence that blood-based biomarkers, used separately or in combination (i.e., panel-based assays), are promising screening tools of AD pathology and for assessing the severity and progression of cognitive decline in patients with MCI.

Most evidence on peripheral biomarkers stems from conventional markers of Aβ and tau; however, research on markers associated with inflammation, oxidative stress, vascular injury, and neurodegeneration is ongoing [43,44,45]. Existing studies of these additional peripheral biomarkers have been limited mostly to cross-sectional examinations of AD. Results among longitudinal studies have been inconsistent, although the strength of evidence generally supports continued development of blood-based biomarkers for clinical use [38,46,47,48,49]. Variability in findings may be due to between-study differences in populations, disease diagnoses, assay methods, biomarkers examined, and study size, among other factors. More research is needed to establish plasma proteins as clinically reliable, predictive, and prognostic biomarkers for MCI and progression to dementia. In particular, additional longitudinal studies involving large diverse populations are needed to elucidate biomarker levels over time, their intraindividual variability, and to understand how other risk factors (e.g., age, sex, APOE genotype, plasma homocysteine) might affect biomarker levels.

Neuroinflammation is a proposed pathogenic contributor to AD and other cognitive disorders [50]. In general, neuroinflammation and abnormal immunologic processes are associated with AD; however, little is known about the progression of these conditions over the disease course [51]. Neuroinflammation is a complex response to brain injury that involves the activation of glia, release of inflammatory mediators such as cytokines and chemokines, and generation of reactive oxygen and nitrogen species that contribute to neuronal cell death [52,53]. Inflammatory responses are complex biological processes that involve multiple mechanisms and cell types. PET is a molecular imaging technique that allows the visualization and quantification of physiological processes by recording the time-dependent distribution of radiolabeled tracer molecules in living organs. Imaging using radioligands specific to active components of the neuroinflammatory process can be used to measure neuroinflammation in cognitive aging and in cognitive disorders. PET radioligands for the 18 kDa translocator protein (TSPO), a mitochondrial protein that is highly expressed in activated microglia, have been developed to assess glial activation and neuroinflammation in vivo [54]. Bioassays have shown increased TSPO binding throughout the brain of AD patients compared to healthy controls, with similar but more modest effects observed in MCI patients [55]. Still, correlations between PET measures and cognition were not present in other studies of MCI patients [51,56]. These findings bring into question whether neuroinflammation is a primary or secondary event in dementia progression. More research is needed to clarify the role of microglial activation in early neurodegeneration (e.g., MCI).

### 3.3. Session III: Neuropathological Changes Associated with Hazardous Exposures and Brain Aging

Session presenters: Dr. Finch, Dr. Spiro III, and Dr. Peskind. Moderator: Dr. Kritikos.

Key Points:

- Exposures potentially associated with neuropathological changes include air pollution and some of its components, toxic emissions from burn pits and other warzone exposures, head trauma, and stress-related disorders (e.g., PTSD).
- Potential neurodegenerative mechanisms associated with air pollution exposures are neuroinflammatory responses, microglial activation, oxidative stress, and disrupted Aβ homeostasis, among others.
- At present, a pathological distinction between dust exposure-induced and stress-induced cognitive decline is not apparent.
- Toxic exposures experienced by World Trade Center responders may well be similar to those experienced by US veterans exposed to burn pits in Iraq and Afghanistan.
- Soldiers with mild traumatic brain injury associated with loss of consciousness also commonly have PTSD, with such co-occurrence reported at 44% in one study.

There is modest heritability of lifespans in humans (15–30%) although some genetic variants may mediate interactions with environmental factors (e.g., alter metabolic pathways) that may increase the risk of age-related disorders. As first argued by Finch and Tanzi (1997), the minor effect and variable penetrance of genetic risk factors on survival and health at advanced ages suggests that lifestyle and environmental factors profoundly influence outcomes of aging [57]. Among environmental factors, exposures that are plausibly associated with neuropathological changes include air pollution and some of its components, toxic emissions from burn pits and other warzone exposures, head trauma, and stress-related disorders (e.g., PTSD). Current data indicate that cognitive decline is characterized by a diverse and complex neuropathologic substrate involving dysfunction of multiple molecular and cellular pathways, resulting in several subtypes of impairment [58]. This heterogeneity may illustrate differing pathomechanistic signatures related to modifiable and non-modifiable risk factors. Still, current data are inadequate to determine how pathology differs by exposure.

#### 3.3.1. Air Pollution

There is mounting evidence from animal and human studies linking air pollution to potential neurological effects [31,59,60,61,62,63]. Based on this evidence, it is estimated that exposure to air pollution contributes approximately 2% of the population attributable fraction for dementia [31]. In general, previous studies have shown that elevated concentrations of nitrogen dioxide (NO_2_), carbon monoxide (CO), nitrous oxides (NO_x_), ozone (O_3_), and ambient particulate matter (PM_2.5_) primarily from traffic exhaust and residential wood burning are associated with increased dementia incidence [59,61]. Neurotoxic particulates and gaseous pollutants may reach the brain directly through circulation (systemic route) or by bypassing the blood–brain barrier altogether via translocation across olfactory epithelium and along olfactory and sensory neuronal pathways (nose-to-brain route) [59,60,63]. Potential neurodegenerative mechanisms include induction of neuroinflammatory responses, microglial activation, oxidative stress, and disruption of Aβ homeostasis, among others [10,61,63,64,65]. Pollutants may also act indirectly through exposure-induced adverse cardiovascular and cerebrovascular effects, which are related to cognitive decline and dementia [66]. It is unknown whether direct and indirect pathways act additively or synergistically. More research is needed to elucidate causal mechanisms and pathways.

To synthesize observational research, Peters et al. (2019) conducted a systematic review of longitudinal studies (minimum of 6 months follow up) that assessed exposure to air pollution and cognitive function in adults [61]. Studies examining tobacco smoke, including passive smoking, or occupational exposures were excluded. That review found 13 studies eligible for synthesis (7 studies examining dementia, 1 AD only, and 6 with cognitive decline measures) that were published from 2012 through September 2018. Study populations were drawn from five countries. Overall, findings among studies supported an association between greater exposure to pollutants, in particular PM_2.5_, NO_2_/NO_x_, and increased risk of dementia. There was consistent evidence that the rate of the exposure-related decline was worse among women and those study participants with the APOE4 allele.

Compared to dementia, the evidence supporting a relationship between air pollution and MCI is much smaller. Positive studies tended to examine populations that were older and less diverse, by both race and sex [12,65], compared to negative studies [67,68]. Given that MCI is subtle relative to dementia an effect from pollution may be masked by other risk factors. Further, cognitive measures have varied between studies, with some focusing on domain-specific cognitive abilities. Gatto et al. (2014) examined healthy, cognitively intact individuals (*n* = 1496, mean age 60.5 years) residing in the Los Angeles Basin and found cross-sectional associations between increasing PM_2.5_ and lower verbal learning; NO_2_ exposure and lower logical memory; and O_3_ exposure and lower executive function [68]. However, there was no evidence of an association between air pollution and the overall composite measure of cognition in that study.

The existing research on air pollution has focused on neurological effects associated with chronic low-level environmental exposure to fine, ultrafine, and nano-sized particles that has occurred over several years. In contrast, the dust and debris cloud from the collapse of the WTC towers and surrounding buildings resulted in an acute exposure to high airborne concentrations of mostly course particles (95% >10 µm), followed by chronic exposures (days to months) to much lower concentrations during recovery and clean-up activities [69]. Nevertheless, the chemical structure of WTC dusts appears sufficient to act as a neurotoxin and researchers have identified several potential pathways linking inhalation exposure to detrimental CNS effects [70]. More research is needed to account for the differences in particle size and exposure profile in order to establish the biologic plausibility of neurologic effects stemming from WTC-related environmental exposure. To date, there is limited epidemiologic evidence supporting an exposure–response association between WTC environmental exposures and cognitive decline. Most studies have been cross sectional, lack an adequate control group, have limited exposure information, and are vulnerable to confounding by unmeasured risk factors. More research is needed to disentangle effects that may be jointly associated with WTC environmental exposures, post-traumatic stress, and other modifiable risk factors.

#### 3.3.2. Warzone Exposures

Gulf War Veterans illness (GWI), is a condition that affects at least one-quarter of the nearly 700,000 veterans who served in the 1990–1991 Persian Gulf War [71,72,73]. It fundamentally differs from trauma and stress-related syndromes described in other wars. Prominent features of GWI are cognitive and psychologic symptoms, including memory loss, confusion, inability to concentrate, depressed mood, and sleep difficulties [71]. Other symptoms include fatigue and musculoskeletal symptoms. Several neurological conditions have been formally evaluated in studies of GW veterans, including amyotrophic lateral sclerosis (ALS), brain cancer, seizures, neuritis and neuralgia, migraine headaches, multiple sclerosis and Parkinson’s disease [72]. These studies have suggested higher rates of brain cancer mortality in GW veterans who were potentially exposed to nerve agents compared to non-deployed controls. Higher rates of repeated seizures, neuralgia/neuritis, migraines, and stroke were also observed in some studies. Mood-cognitive problems are among the most prevalent self-reported symptoms among Gulf War (GW) veterans [73,74]. A recent meta-analysis of 14 studies published from 1992 through May 2015 that reported on neuropsychological performance in GW veterans found significantly decreased performance in three functional domains: attention and executive function, visuospatial skills, and learning/memory [73]. Deployment-related PTSD and other psychiatric conditions have also been studied. Population-based studies suggest rates of PTSD in GW veterans of less than 6%; however, much higher rates were found among veterans with GWI [72].

Neurotoxic exposures that appear to be more strongly associated with GWI are pyridostigmine bromide pills that were given to protect troops from the effects of organophosphate nerve agents, and pesticide use during deployment (e.g., insecticide-treated uniforms, insecticide-treated tents, heavy use of pesticide sprays, bug strips, and wearing flea collars around the neck and ankles). Other potential exposures that are suspected to be involved include low-level exposure to nerve agents, proximity to oil well fires, simultaneous receipt of multiple vaccines, and synergism caused by combining these Gulf War exposures. In a cross-sectional study, chemical warfare-exposed Gulf War veterans had reduced gray matter and white matter volumes compared to unexposed veterans [75]. Studies of GWI are limited by the fact that there are no objective diagnostic criteria for the illness, such as laboratory abnormalities or characteristic physical signs, so diagnosis is a matter of excluding other causes of the symptoms.

Another source of potential neurotoxic exposure stems from open-air burn pits used for solid waste disposal during military operations in Iraq and Afghanistan [76,77]. The wastes were ignited using various accelerants (e.g., JP-8 jet fuel), and other substances. Potential smoke hazards associated with burn pits include burning plastic, polystyrene, paper, wood, rubber, petroleum oil and lubricants, non-medical waste, metals, paints and solvents, and incomplete combustion of byproducts. Emissions from the pits may include particulates, dioxins, furans, lead, mercury, volatile organic compounds, and PAHs, among other hazardous substances [78,79]. The toxic exposures experienced by WTC responders and survivors may be similar to those experienced by veterans exposed to burn pits.

Other stressors potentially affecting cognition in veterans are military lifestyle issues, alcohol misuse, body building supplements, hyperthermia and dehydration in the blistering heat of the Iraq warzone, relative hypoxia due to the altitude in the Afghanistan warzone (i.e., Kabul is 5900 feet above sea level), the chronic stress and misery of living in the warzone, and chronic sleep deprivation.

#### 3.3.3. Head Trauma

Common causes of mild traumatic brain injury (mTBI) include exposure to blasts (e.g., improvised explosive device or artillery) among military personnel and veterans and head trauma experienced by semiprofessional and professional athletes. In addition to the acute symptoms of headache, fatigue, depression, anxiety, irritability, and transient impaired cognitive function, mTBI can also lead to chronic neurocognitive effects. Chronic neurocognitive effects are more common among those who experienced repetitive mTBIs versus a single mTBI [80].

It is estimated that among soldiers who served in the Iraq warzone, approximately 15% experienced mTBI [80]. In addition, 44% of soldiers with mTBI associated with loss of consciousness also had PTSD. Another study among soldiers who experienced blast mTBI while deployed to Iraq or Afghanistan on average had a total of 20 blast mTBI events during their entire military service [81]. In studies of veterans, there has been a lack of consistency in the anatomic effects of blast mTBI. This may be related to the biomechanics of brain injuries resulting from explosive blast waves, which can create complex shear force patterns which may lead to spatially heterogeneous patterns of injury across individuals [81].

#### 3.3.4. PTSD

The American Psychiatric Association’s Diagnostic and Statistical Manual, fifth edition (DSM-V5) describes PTSD as a set of symptoms including intrusive and distressing thoughts and feelings that can follow exposure to a traumatic event (e.g., war, disaster, sexual assault), where these symptoms cause marked impairment that is still present one or more months after the trauma [82]. PTSD imposes a considerable health burden, with national lifetime, past 12 month, and past 6 month prevalence estimated at 8.3%, 4.7%, and 3.8%, respectively [83]. In studies of highly exposed first responders, cross-sectional prevalence of PTSD symptoms have varied, ranging from 8% at 7.5 months post-9/11 to 23% within two weeks of exposure [84]. PTSD prevalence also varied by occupation. In a large study of rescue and recovery workers enrolled in the World Trade Center Health Registry (*n* = 28,962), the overall prevalence of PTSD was 12.4%, ranging from 6.2% for police to 21.2% for unaffiliated volunteers, suggesting that risk was greatest among those without prior disaster training or experience [85].

There is considerable literature supporting a relationship between chronic PTSD and impaired cognitive function, mostly among war veterans and survivors [7,8,86] and persons exposed to disasters [1,2,3,5,87]. Still, it is difficult to conclude that PTSD, a highly comorbid condition, is the primary cause of cognitive impairment observed in most individuals. For example, there is a frequent association between mTBI and PTSD among veterans. There can also be considerable overlap among post-concussive syndrome, chronic traumatic encephalopathy, and PTSD in veterans and disaster populations; all of which are characterized by neuropsychiatric symptoms [88,89]. Other potential causal factors for cognitive impairment that can result from PTSD include sleep deprivation, sleep apnea, cardiovascular risk factors (e.g., smoking, hypertension, hypercholesterolemia, diabetes), among others. The complexity caused by interrelations among multiple risk factors makes it difficult to disentangle causal pathways involving PTSD and cognitive impairment.

#### 3.3.5. Biological Aging

It is well known that there is considerable population variation in the rate at which individuals age and the rate at which frailty and disease appear [90]. This concept of variation in rates of aging in often referred to as “biological” aging. Objective measurement of biological aging may allow a better understanding of suspected risk factors and the identification of effective health-promoting interventions, perhaps in a personalized and disease-specific fashion. One tool to measure biological aging is the ‘epigenetic clock’, a DNA methylation-based predictor that is comprised of 353 cytosine-phosphate-guanosine sites (CpGs) across the genome [91]. Mentioned at the workshop were some sources of data on MCI and dementia that perhaps can be used as control/comparison groups for 9/11-exposed populations. These included Psychiatric Genetics Consortium on PTSD (https://pgc-ptsd.com/); CAP—the Consortium to Alleviate PTSD; and, IALSA, the Integrative Analysis of Longitudinal Studies of Aging and Dementia (https://www.ialsa.org/).

### 3.4. Session IV: Cognitive Decline and Impairment in the 9/11-Exposed Population

Session presenters: Dr. Clouston, Dr. Hall, and Dr. DeKosky. Moderator: Dr. Bromet.

Key Points:

- The 9/11-affected population is entering a time when aging is becoming more clinically significant.
- Epidemiologic data suggest an increased risk of cognitive impairment and functional limitations among persons directly affected by the terrorist attacks. However, most studies have been cross sectional and thus provide limited information on cause and effect.
- Mechanisms of WTC-related cognitive impairment remain elusive. A suggested mode of action is through PTSD and/or major depression. Exposure to WTC dust and particulate matter has also been suggested; however, there is less evidence supporting this pathway.
- Neuroimaging of WTC responders with cognitive impairment revealed patterns of reduced cortical thickness that were largely inconsistent with those for known neurodegenerative diseases.
- Research clarifying the causal pathway(s) may lead to more effective interventions and improvements in patient care.

This session summarized existing literature on MCI in the 9/11-exposed population. The literature consisted mainly of cross-sectional studies of firefighters and emergency medical staff or general responders employed in law enforcement who were exposed during and following the WTC attacks. The workshop focused on the evidence of an effect (i.e., strength of evidence) rather than significant study limitations, which is reflected in the following summary.

#### 3.4.1. General Responder Studies

There have been several investigations examining cognitive impairment and functional limitations in WTC general responders who were mostly male, white, middle aged, college educated, and in law enforcement in 2001. The first study evaluated the association of PTSD and major depressive disorders (MDDs) with cognitive impairment in a sample of responders (*n* = 813) without concurrent head trauma [1]. Study participants were screened for cognitive impairment and dementia using the Montreal Cognitive Assessment (MoCA) from January 2014 through April 2015. Concurrent diagnoses of PTSD, and MDD, and inventories measuring PTSD and depressive symptoms, were collected annually since 2002. This study found that 12.8% of participants had MoCA scores indicating cognitive impairment, and approximately 1.2% suggesting dementia. Further, current PTSD (adjusted RR = 1.9, 95% CI: 1.2, 2.8) and MDD (adjusted RR = 2.2, 95% CI: 1.3, 2.8) were associated with cognitive impairment after adjustment for predisposing characteristics, WTC exposures, and health and behavioral mediators. The APOE4 allele was associated with increased risk of possible dementia, but it was not associated with risk of PTSD and it did not modify the association between WTC exposure and cognitive impairment.

A subsequent study evaluated whether WTC exposures and PTSD were associated with overall cognitive dysfunction in general responders and with cognitive domains implicated in neurodegeneration [3]. This study compared the cognitive performance of responders with no history of stroke or head injuries (*n* = 1193) to normative data from age-matched cognitively healthy adults. Cognitive functioning was assessed between November 2015 and June 2016 via Cogstate (www.cogstate.com), which is a computer-assisted neuropsychological battery measuring three domains (reaction speed, processing speed, and memory) and three subdomains (attention, response variability, and cognitive efficiency) of cognition [92,93,94,95,96]. The average age at test administration was 53.7 years. The study found that 14.8% of participants had cognitive dysfunction compared to normative data, and that PTSD symptom severity and working more than 5 weeks on site were associated with lower cognition. Additionally, WTC responders without PTSD showed slower reaction speed, processing speed, and worse memory, compared to normative data.

A sample of general responders (*n* = 1800) who were cognitively normal at baseline was followed for 18 months. Of these, 255 (14.2%) presented with MCI during follow up for an age-standardized incidence rate per 1000 person-years of 159.5 (95% CI: 154.8, 162.2). Based on sparsely published rates in the general population, MCI incidence among responders appeared greater than expected (SIR = 2.16, 95% CI: 1.92, 2.41). Multivariable regression modeling revealed that MCI incidence was highest in responders with increased PTSD symptom severity compared to those experiencing low severity (HR = 2.67, 95% CI: 1.33, 5.37). Prolonged WTC exposure, defined as working on the debris pile for 15 weeks or more, was associated with MCI in responders with the APOE4 allele (HR = 3.89, 95% CI: 1.79, 8.46); however, there was no evidence of an exposure–response association between MCI and prolonged exposure (HR = 0.93, 95% CI: 0.55, 1.55) or between MCI and APOE4 (HR = 1.04, 95% CI: 0.64, 1.65). when analyzed separately [5].

A cross-sectional study of 1268 responders found that approximately 16% presented with functional limitations (e.g., walking speed, chair rise times), and that PTSD was associated with a two-fold increased risk of these limitations [97]. Another cross-sectional study of 2016 WTC responders showed significantly lower mean hand grip strength (HGS) among responders with both WTC-related PTSD and depression compared to those with those who had WTC-related PTSD, but not depression [98]. Re-experiencing symptoms, a PTSD domain, was associated with both cognitive impairment and lower HGS [97,98].

##### Biomarker and Neuroimaging Studies

Given its association with cognitive impairment and neurodegeneration, PTSD gene expression profiling may elucidate causal mechanisms. There have been two PTSD gene profiling studies of WTC responders [99,100]. Whole-blood RNA analyses of 324 WTC responders with and without PTSD identified five genes (FKBP5, NDUFA1, CCDC85B, SNORD54, SNORD46) that are differentially expressed in those with PTSD [99]. NDUFA1 expression has also been associated with Parkinson’s disease, AD and Huntington’s disease. A subsequent study of 39 WTC responders (20 with and 19 without PTSD) characterized the gene regulation of PTSD by profiling the gene expression of four immune cell subpopulations (CD4T, CD8T, B cells, and monocytes) in 39 WTC responders with and without PTSD [100]. The largest differences in PTSD gene expression were identified in monocytes, which play a role in the process of adaptive immunity.

A cohort study of 398 WTC responders applied the AT(N) model [26] using plasma-based markers for Aβ_42_ and Aβ_40_ for Aβ (A), total tau for tauopathy (T), and NfL for neurodegeneration (N) [101]. This study built upon a pilot study of 34 WTC responders without concomitant head injury that showed an association between increased PTSD and decreased plasma Aβ load and increased Ab42/Ab40 ratios [4]. Results from the larger study [101] showed that WTC responders with cognitive impairment had higher age, lower raw mean plasma levels of Aβ_42_ and a higher ratio of Aβ_40_:Aβ_42_. These findings support the AT(N) pathways and showed that Aβ_40_ plays an intermediary role linking the proliferation of tau with changes in neurodegeneration.

A proteomics study evaluated 276 proteins with known involvement in PTSD and MCI in a sample of WTC responders (*n* = 181) without history of medical or neurodegenerative conditions, brain tumors, cancers, or cerebrovascular conditions. The sample included 34 responders with comorbid PTSD-MCI, 39 with PTSD only, 27 with MCI only, and 81 unaffected controls. Probable PTSD and MCI were measured by Post-Traumatic Stress Disorder Checklist-Specific Version (PCL-17) and MoCA, respectively. The study identified 50 proteins that could serve as potential protein biomarkers for PTSD, MCI and comorbid PTSD-MCI [102]. Of these, 16 were associated with comorbid PTSD-MCI, 24 with PTSD-only, and 20 proteins with MCI-only. Two proteins, neurocan (NCAN) and brevican (BCAN), appeared most promising as potential biomarkers for measuring disease burden. Results from the multiprotein composite scores showed higher discerning power for MCI only and comorbid PTSD-MCI compared to PTSD only.

Neuroimaging studies were conducted within 4 years of the attacks to evaluate the effects of 9/11-related psychological trauma exposure on the brains of healthy adults who were within 1.5 miles of the disaster site compared to controls who were at least 200 miles away [103,104]. The largest study examined 34 adults (17 exposed, 17 controls). In that study, there were no significant differences between groups in terms of age, sex, age at first trauma, years since most recent trauma, number of traumas in lifetime, number of traumas in lifetime with shock, terror, or horror, or past history of PTSD. The exposed group experienced their worst trauma more recently than the comparison group (*p* = 0.001). Neither group included persons diagnosed with PTSD; however, the exposed group reported a higher number of current PTSD symptoms (*p* = 0.02) and lower gray matter volume in amygdala, hippocampus, insula, anterior cingulate, and medial prefrontal cortex. These findings suggested that trauma exposure may play a causal role in changes to brain structure and function. A later cross-sectional neuroimaging study found that whole-brain and regional cortical thickness (CTX) was reduced in responders with cognitive impairment (*n* = 51) compared to unimpaired responders in analyses controlled for age at scan, sex, race/ethnicity, occupation, and educational attainment. Cognitive impairment was measured by clinicians using the MoCA. PTSD status, attained by trained psychologists using the Structured Clinical Interview for the Diagnostic and Statistical Manual of Mental Disorders-IV (SCID-IV) [105], did not predict reduced CTX across groups [106]. Both cognitively impaired and unimpaired groups had reduced CTX in the entorhinal and temporal cortices compared to published normative data. There is emerging evidence that these regions are vulnerable to exposure to inhaled neurotoxins [107]. The authors noted that reduced CTX and volume of the entorhinal cortex is often interpreted as an early marker of the spread of tauopathy in AD [106]. Patterns of CTX reduction were most similar to those found in an uncommon parietal-dominant subtype of AD and related dementias. Still, these patterns were largely inconsistent with those for known neurodegenerative diseases, which was interpreted by the authors as suggestive of an encephalopathy of unknown etiology that is characterized by widespread cortical atrophy.

#### 3.4.2. FDNY Firefighter/EMS Studies

There have been similar findings in recent studies of firefighters and emergency medical service (EMS) workers employed by the Fire Department of the City of New York (FDNY) who were actively employed on 9/11, arrived at the WTC site between 11 and 24 September 2001, and had regular physical and mental health monitoring exams. Singh et al. (2020) examined FDNY workers (*n* = 7875) who completed a self-administered subjective measure of cognition and function, the Cognitive Function Instrument (CFI), between 1 March 2018 and 28 February 2019. The mean age at CFI completion was 57 years. The most common cognitive concerns were memory related. Participants with high-intensity WTC exposure had an increased likelihood of top-quartile CFI score indicating greater impairment, with an odds ratio (OR) of high versus low exposure of 1.32, (95% CI: 1.07, 1.64). Current and early probable PTSD were both associated with top-quartile CFI (OR = 3.25, 95% CI: 2.53, 4.19 and OR = 1.56, 95% CI: 1.26, 1.93), respectively [2]. A subsequent cross-sectional study further examined the interrelationships among probable PTSD, self-reported depressive symptoms, environmental exposure, and cognitive decline in FDNY workers (*n* = 9516) [87]. This study fit multivariable regression models that allowed for mediation of WTC exposure by PTSD or depressive symptoms. Exposure intensity was expressed in categories (high, moderate, and low) based on each worker’s arrival time at the WTC site. Exposure–response models controlled for age, sex, race, education level, work assignment on 9/11, and smoking status. The study found that PTSD symptoms completely mediated the association between WTC exposure level and self-reported cognitive decline. Depressive symptoms were also a significant mediator between WTC exposure and cognitive decline. The study was not able to fully address the relative importance of depressive symptoms vs. PTSD symptoms as mediators because of a high mutual comorbidity between the two. Moreover, study findings merit cautious interpretation given the cross-sectional design and subjective cognitive measure.

#### 3.4.3. WTC Health Registry Enrollees

There are over 71,000 responders and community members enrolled in the WTC Health Registry. Four major surveys have examined enrollees’ exposures to the disaster, and short- and long-term health effects (wave 1, 2003–2004; wave 2, 2006–2007; wave 3, 2011–2012; and wave 4, 2015–2016). One study examined the incidence of self-reported confusion and memory loss in a sample of 14,574 enrollees who completed wave 3 and 4 surveys and who were aged 35–64 years at time of wave 4 [108]. Those with history of stroke or who reported confusion or memory loss at wave 3 were excluded. The majority (62%) of the sample was male and aged 55–64 years. PTSD status was determined by self-report using the total score from the Post-Traumatic Stress Disorder Checklist scale. Approximately 22% of enrollees self-reported confusion or memory loss at the wave 4. Enrollees with higher educational attainment, increased social support (engaging with others and feeling supported by others), and greater levels of physical and mental activity were less likely to report confusion or memory loss, regardless of probable PTSD status. However, these effects were stronger in the group with non-probable PTSD.

### 3.5. Session V: Clinical Perspectives

Session presenters: Dr. Sloan, Dr. Raskind, and Dr. Fausto. Moderator: Dr. Diminich.

Key Points:

- There are currently no proven interventions to reduce the risk, delay the onset, or slow the progression of MCI.
- Optimization of the management of associated comorbidities, including PTSD and hypertension, may be important in reducing the risk of MCI.
- Lifestyle modifications are proven strategies to improve overall well-being, health, and aging.
- Cognitive training is a promising tool for prevention and treatment of MCI and dementia, with clinically meaningful effects beyond cognition including other aspects of well-being.

Prevention through management of known risk factors is the primary defense against the onset of MCI and dementia [31]. Risk in the WTCHP population might be reduced through effective care coordination with primary care providers and enhancement of health strategies and policies that:

- Reduce hypertension risk in the entire population.
- Encourage social, cognitive, and physical activity.
- Scrutinize the risks for hearing loss throughout the life course of WTCHP members.
- Reduce tobacco and alcohol use.

Prevention activities that target WTCHP members may include:

- Treating hypertension and aim for systolic blood pressure <130 mm Hg in midlife;
- Promoting the use of hearing aids for hearing loss;
- Avoiding drinking 21 or more units of alcohol per week;
- Preventing head trauma;
- Smoking cessation;
- Reducing obesity and the linked condition of diabetes by healthy food availability;
- Sustaining midlife, and late-life physical activity.

Not all cognitive dysfunction can be prevented; therefore, post-diagnosis interventions and care are needed. Interventions are intended to inhibit the progression of MCI to dementia and involve several approaches to decrease further neuropathological damage (treating vascular risk factors, diabetes, diet, exercise), combined with maximizing function via cognitive and social stimulation, as well as treatment of neuropsychiatric symptoms [24]. Currently, there are no approved medications for MCI risk reduction. The evidence from existing studies of non-pharmacological interventions is also equivocal [109,110]. Yet, new studies aimed to prevent the onset or mitigate the course of cognitive decline are emerging. Many of these studies have focused on lifestyle modifications, alternative pharmaceuticals, and cognitive training.

#### 3.5.1. Lifestyle Modifications

Lifestyle modifications have important salutary effects on significant chronic diseases, including mental health disorders, diabetes, and disability. Physical activity, including aerobic exercise, is beneficial for healthy aging, and early intervention may prevent, or delay age-related global declines in health. There is a growing literature showing positive effects of exercise on cognitive functioning. Most systematic reviews report modest beneficial effects of physical activity on a wide range of cognitive functions across the life course [111,112,113,114,115,116]. The strongest evidence stems from studies showing improvements in processing speed, memory, and executive function in children aged 6–13 years and adults aged >50 years [116]. Still other reviews have found limited evidence supporting an effect [117,118,119]. Inconsistencies in the literature are likely due in part to a relatively small number of high-quality studies, variability in study populations, and heterogeneity in study designs involving evaluations of different cognitive domains and exercise protocols. More information is needed on interactions between cognitive and physical interventions. Furthermore, few studies provide information on the long-term effects of exercise on cognitive function and the sustainability of the positive effects observed [115]. Nearly all reviews call for additional well-designed studies to explore the potential benefits of exercise on cognition in support of recommending interventions.

Identifying mechanisms remains important to elucidate specific targets that produce positive cognitive benefits. It is likely that positive effects are attributable to multiple mechanisms that vary widely between individuals, yet there may be some sharing across certain groups. One hypothesis suggests the cognitive effects of exercising are largely mediated through circulating inflammatory markers. These biomarkers are the subject of many vascular studies, which have implications for cognitive aging given potentially similar pathophysiologic mechanisms. For example, the CANTOS trial found that high levels of interleukin-6 (IL-6) were significantly reduced by 150 mg of canakinumab (a human anti-IL-1β monoclonal antibody) administered subcutaneously every 3 months, with the risk of the key secondary cardiovascular end point 17% lower than that in the placebo group (4.29 vs. 5.13 events per 100 person-years) [120]. Observational measurement in other studies of this and other markers demonstrate cardioprotective effects as well, but interventional or provoked studies reveal mixed effects depending on methodology and have also reported an increase in pro-inflammatory factors such as IL-6 [121]. One study that enrolled 119 individuals randomized to twelve weeks of training versus sedentary behaviors yielded the expected significant increase in aerobic capacity in the training but not the wait-list control condition, and post-hoc analysis revealed a training-induced enhanced IL-6 and TNF-α response to ex vivo stimulation, a finding suggestive of a proinflammatory response [121]. A clear understanding of the role of inflammatory markers in cognition will help develop therapeutics and interventions to achieve targets, including exercise inducible targets, to combat age related decline.

#### 3.5.2. Pharmacologic Interventions

Cholinesterase inhibitors, which are approved for the symptomatic treatment of mild to moderate AD, have been of interest for MCI treatment, but numerous studies have failed to indicate a benefit in reducing MCI or the progression to dementia [109,122]. Therefore, other medications are being analyzed in clinical trials (NIH/NIA). Primary outcomes of these trials include not only MCI, but some trials offer novel therapies that address risk factors for the disease, including PTSD and hypertension.

PTSD pathophysiology, especially disruptive nocturnal symptoms, has been associated with increased brain norepinephrine in combat veterans. Prazosin is an alpha-1 adrenergic receptor (AR) antagonist that crosses the blood brain barrier, reducing noradrenergic activity. A series of randomized clinical trials in military have demonstrated positive clinical effects of Prazosin, including reduced PTSD symptoms, decreased nightmare frequency, and improved sleep quality [123]. A subgroup analysis showed participants with higher pre-treatment blood pressures (BP) had greater PTSD symptom reduction; for every 10 mm Hg increase in pretreatment BP, there was an additional 14 point reduction in total CAPS (Clinician-Administered PTSD Scale) score (*p* = 0.002) [124].

The recent Systolic Blood Pressure Intervention Trial—Memory and Cognition in Decreased Hypertension (SPRINT MIND) study examined the relationship between aggressive treatment of hypertension and the development of MCI. In 9361 hypertensive older adults with increased cardiovascular risk (but without diagnosed diabetes, dementia or stroke), intensive BP control to a goal of 120 mmHg significantly reduced the risk of mild cognitive impairment (14.6 vs. 18.3 cases per 1000 person-years; HR = 0.81; 95% CI: 0.69, 0.95) during 5 years of follow up, but not the development of dementia (7.2 vs. 8.6 cases per 1000 person-years; HR = 0.83; 95% CI: 0.67, 1.04) [125].

#### 3.5.3. Cognitive Training

Processes to exercise the brain to prevent or treat dementia represent a substantial research domain. However, results are mixed, possibly due to the variabilities in the cognitive functions that were assessed. Useful Field of View (UFOV), or speed of processing training, is an adaptive visual training and assessment technology, applied to enhancing cognitive functions. A recent systematic review and meta-analyses of 44 published studies from 17 different randomized clinical trials published in peer-reviewed journal articles indicated that UFOV training has multiple benefits, including positive effects in attention, daily functioning, and well-being [126]. The Advanced Cognitive Training for Independent and Vital Elderly (ACTIVE) trial randomized 2832 older adult participants to 10 or more sessions of training, performed baseline neuropsychological testing, and then followed participants over 10 years to assess longitudinal cognitive benefits. Intervention participants reported less difficulty with memory, reasoning, and speed of processing [127]. Additionally, participants randomized to speed of processing cognitive training had a 29% reduction in dementia (HR 0.71, 95% CI: 0.50, 0.998, *p* = 0.049) [128]. Further studies are required to identify the specific aspects of these interventions that produce global benefits.

### 3.6. Session VI: Monitoring and Surveillance

Session presenters: Dr. Roberts and Dr. Reissman. Moderator: Dr. Luft.

A short listening session was held to exchange information on monitoring and surveillance practices. The session focused on current practices within the WTCHP and at other institutions. Participants discussed the importance of identifying potentially high risk subgroups as a potential focus for initial monitoring initiatives, such as individuals with high exposure to 9/11 particulate during recovery and rehabilitation efforts and those with PTSD. Gathering data longitudinally is preferred, such as repeat measures using a combination of clinical interviews, informant (family/guardian) reports, and global screening instruments, among others, when mild impairment is suspected. Brief screening instruments that include measures of both executive function (e.g., planning, organizing), and episodic memory (e.g., recollection of specific events, situations, and experiences) were preferred. Some workshop participants suggested that routine cognitive monitoring include brief physical performance assessments (e.g., a timed walk) in concert with global screening instruments. It was also suggested that adding a measure of anxiety should be considered given its relative importance to CVD, cognition, and PTSD.

## 4. Discussion

The 9/11-exposed population is entering an age in which cognitive dysfunction is becoming more clinically relevant. There is growing evidence suggesting that 9/11-exposed first responders are at increased risk of MCI and other neurological deficits, including early signs of neurocognitive and neuromotor dysfunction typically seen at older ages. This existing evidence is strongest for a potential link between chronic PTSD and MCI risk, although environmental exposure to dust and particles that resulted from the collapse of the towers and subsequent cleanup activities cannot be ruled out as a contributing cause. However, this evidence stems mainly from observational studies that are subject to several limitations, such as selection bias, statistical power, and measurement error. Many of the studies are cross sectional; therefore, they can provide little information on cause and effect. Some studies relied on subjective measures of cognition, which are prone to bias. Moreover, existing findings stem primarily from examinations of general responders; therefore, replication of findings in larger, more heterogeneous samples of 9/11-exposed populations is still needed to understand how MCI risk differs by factors such as sex, race/ethnicity, and socioeconomic status. The pathology and mechanisms of MCI are complex and poorly elucidated, which may explain its heterogenous clinical presentation and general lack of treatment options. Research on biomarkers of effect, such as further studies using proteomic and metabolomic approaches, may further elucidate causal mechanisms. In summary, more research is needed to fully characterize the health burden in the affected population and develop effective prevention and treatment strategies.

The workshop described herein was convened to exchange information on the current science of MCI and catalogue individual perceptions on future research needs. The workshop stimulated collaboration and initiated new ideas that, along with input from other scientists and stakeholders, will be useful in planning research on cognitive decline and other issues related to aging in the 9/11-exposed population. To build research capacity, the WTCHP is pursuing an agenda that targets a broad range of studies that characterize the health burden and inform treatment. The research goals are to maximize available resources through collaboration, improve the understanding of the risk and underlying etiology of cognitive impairment among WTCHP members, and to develop risk-reduction and mitigation strategies. Future research projects will address important limitations that have been observed in existing studies, such as inconsistent definitions of cognitive impairment, inadequate information characterizing baseline risks, exposure misclassification, and a lack of non-exposed comparison groups. Additionally, continued advancement in neuroimaging and biomarker research offer potential improvements in surveillance and diagnostic cost and capability. Similarly, advances in molecular sciences (e.g., genomics, epigenomics, proteomics) may uncover mechanistic data that elucidate causal pathways and possibly lead to better care. Specific areas of interest include:

- *Cognitive Resilience and Impairment:* Projects that describe the characteristic features of cognitive impairment in 9/11-exposed individuals and its progression and identify factors that influence the maintenance of cognitive performance or cognitive resilience in the face of environmental exposure and psychological trauma.
- *Early Identification of Cognitive Decline:* Projects that examine changes in non-cognitive domains (e.g., gait, audition, emotional expression, reactivity, and olfaction) that may serve as early markers of neurodegeneration.
- *Pathophysiology of Cognitive Decline:* Projects that focus on the neurobiological mechanisms underlying shifts in the nature and progression (trajectories) of age-related cognitive decline or cognitive impairment.
- *Impact of Life Stressors on Cognition:* Studies of association between individual and combined childhood and adulthood life stressors (adversity) on cognition in aging.
- *Alzheimer’s Disease:* Projects that examine the impact and trajectories of Alzheimer’s disease or Alzheimer’s disease-related dementias (AD/ADRD) among WTCHP members.

The agenda will continue to promote collaboration that expands capabilities of research on aging and cognition. For example, the WTCHP and the National Institute on Aging (NIA) have developed a joint funding solicitation to support exploratory and developmental research projects related to cognitive aging and impairment in the 9/11-exposed population. Future collaborations may include other joint funding opportunities or support data collection from multiple sources of patient information on various health risks, including cognitive impairment. For example, researchers are currently exploring linkages with existing large state health databases that may contain information related to cognitive function and mental health on unexposed populations that may be suitable for use as comparison groups.

## 5. Conclusions

The James Zadroga 9/11 Health and Compensation Act of 2010 (Zadroga Act), and its reauthorization in 2015, established a program through 2090 to monitor and treat responders and survivors suffering from adverse health conditions attributable to the 9/11 attacks. As the affected population enters midlife, there is growing evidence of an increased risk of cognitive decline. The WTCHP is committed to improving its research agenda to support the care and well-being of its members. A scientific workshop on MCI was convened in 2019 to begin framing program initiatives that will help characterize the cognitive health burden and inform treatment under the WTCHP. Workshop participants, experts in the area of cognitive aging and impairment, described the state of the science in six listening sessions spanning two days. The exchange of facts, information, and individual perspectives will help in planning future research on cognitive decline and other issues related to aging in the 9/11-exposed population.

## Figures and Tables

**Table 1:** Workshop listening sessions and discussion questions.

Session I: The Natural History of Cognitive Aging and Impairment
1. What is the natural history of brain structure and function later in life (i.e., what occurs as individuals age)?
2. What do we mean by cognitive impairment (narrowing our scope)?
3. If you have MCI, what are the potential consequences of this (prognosis), especially with respect to debilitating disease like dementia?
4. Other than aging, what are the predictors (risk factors) and causes of cognitive impairment, including nosology of vascular and neurodegenerative diseases and cognitive effects of medications used to treat other conditions?
Session II: Novel Identifiable Markers in the Pathway of Neurodegenerative Disease
5. What is the histopathology of MCI?
6. What test(s) are the most promising at this point and that may be relevant for the 9/11-exposed population?
7. What test(s) are the most sensitive and specific for clarifying disease diagnosis?
Session III: Neuropathological Changes Associated with Hazardous Exposures and Brain Aging
8. What types of agents are likely associated with neuropathological changes?
9. What is the biologic plausibility for 9/11 agents to cause neuropathological change (mechanisms)?
10. Is there a pathological distinction between dust exposure-induced and stress-induced MCI?
Session IV: Cognitive Decline and Impairment in the 9/11-Exposed Populations
11. Review of research (completed and current) involving the 9/11-exposed population.
12. Is there a 9/11 phenotype, and how common are symptoms?
13. Characterize who in the cohort is affected?
14. Is it primarily a subset of individuals with PTSD?
15. Are WTCHP members at increased risk for MCI?
16. What are the predictors of MCI among WTCHP members?
17. Address pathway modeling for 9/11 exposures and MCI, if possible.
Session V: Clinical Perspectives on Aspects and Targets for Treatment
18. How do we best characterize and treat MCI with and without psychiatric comorbidity?
19. What are the most informative neurocognitive tests to detect and measure the course of MCI?
20. Can MCI be prevented, or can decline in cognitive functioning be slowed?
21. What are patient eligibility criteria for ongoing clinical trials?
Session VI: Monitoring and Surveillance
22. Role of screening for MCI in monitoring or using a subset of the cohort—and which tool?
23. What (baseline) testing should be performed now that could be of value when doing longitudinal follow up?

**Table 2:** NIA-AA biomarker grouping scheme (adapted from Jack et al. [26]).

A: Aggregated Aβ or Associated Pathologic State
CSF Aβ_42_, or Aβ_42_/Aβ_40_ ratio
Amyloid PET
T: Aggregated tau (neurofibrillary tangles) or associated pathologic state
CSF phosphorylated tau
Tau PET
(N): Neurodegeneration or neuronal injury
Anatomic MRI
FDG PET
CSF total tau
CSF NfL

Abbreviations: AA, Alzheimer’s Association; Aβ, β amyloid; CSF, cerebrospinal fluid; FDG, fluorodeoxyglucose; MRI, magnetic resonance imaging; NfL, neurofilament light chain protein; NIA, National Institute on Aging; PET, positron emission tomography.

## Data Availability

No new data were created or analyzed in this study. Data sharing is not applicable to this article.
